# Genetically Predicted Blood Pressure and Risk of Atrial Fibrillation

**DOI:** 10.1161/HYPERTENSIONAHA.120.16191

**Published:** 2021-01-04

**Authors:** Matthew C. Hyman, Michael G. Levin, Dipender Gill, Venexia M. Walker, Marios K. Georgakis, Neil M. Davies, Francis E. Marchlinski, Scott M. Damrauer

**Affiliations:** 1 From the Division of Cardiovascular Medicine (M.C.H., M.G.L., F.E.M.), University of Pennsylvania Perelman School of Medicine, Philadelphia; 2 Department of Surgery (V.M.W., S.M.D.), University of Pennsylvania Perelman School of Medicine, Philadelphia; 3 Corporal Michael J. Crescenz VA Medical Center, Philadelphia, PA (M.G.L., S.M.D.); 4 Department of Epidemiology and Biostatistics, School of Public Health (D.G.), Imperial College London, United Kingdom; 5 Department of Medicine, Centre for Pharmacology and Therapeutics, Hammersmith Campus (D.G.), Imperial College London, United Kingdom; 6 Department of Genetics, Novo Nordisk Research Centre Oxford, Old Road Campus, United Kingdom (D.G.); 7 Clinical Pharmacology and Therapeutics Section, Institute of Medical and Biomedical Education and Institute for Infection and Immunity, St George’s, University of London, United Kingdom (D.G.); 8 Clinical Pharmacology Group, Pharmacy and Medicines Directorate, St George’s University Hospitals NHS Foundation Trust, London, United Kingdom (D.G.); 9 Medical Research Council Integrative Epidemiology Unit (V.M.W., N.D.), University of Bristol, United Kingdom; 10 Bristol Medical School: Population Health Sciences (V.M.W.), University of Bristol, United Kingdom; 11 Institute for Stroke and Dementia Research, University Hospital, Ludwig-Maximilians-University LMU, Munich, Germany (M.K.G.).

**Keywords:** atrial fibrillation, blood pressure, calcium channel blockers, hypertension

## Abstract

Observational studies have shown an association between hypertension and atrial fibrillation (AF). Aggressive blood pressure management in patients with known AF reduces overall arrhythmia burden, but it remains unclear whether hypertension is causative for AF. To address this question, this study explored the relationship between genetic predictors of blood pressure and risk of AF. We secondarily explored the relationship between genetically proxied use of antihypertensive drugs and risk of AF. Two-sample Mendelian randomization was performed using an inverse-variance weighted meta-analysis with weighted median Mendelian randomization and Egger intercept tests performed as sensitivity analyses. Summary statistics for systolic blood pressure, diastolic blood pressure, and pulse pressure were obtained from the International Consortium of Blood Pressure and the UK Biobank discovery analysis and AF from the 2018 Atrial Fibrillation Genetics Consortium multiethnic genome-wide association studies. Increases in genetically proxied systolic blood pressure, diastolic blood pressure, or pulse pressure by 10 mm Hg were associated with increased odds of AF (systolic blood pressure: odds ratio [OR], 1.17 [95% CI, 1.11–1.22]; *P*=1×10^−11^; diastolic blood pressure: OR, 1.25 [95% CI, 1.16–1.35]; *P*=3×10^−8^; pulse pressure: OR, 1.1 [95% CI, 1.0–1.2]; *P*=0.05). Decreases in systolic blood pressure by 10 mm Hg estimated by genetic proxies of antihypertensive medications showed calcium channel blockers (OR, 0.66 [95% CI, 0.57–0.76]; *P*=8×10^−9^) and β-blockers (OR, 0.61 [95% CI, 0.46–0.81]; *P*=6×10^−4^) decreased the risk of AF. Blood pressure–increasing genetic variants were associated with increased risk of AF, consistent with a causal relationship between blood pressure and AF. These data support the concept that blood pressure reduction with calcium channel blockade or β-blockade could reduce the risk of AF.

## Abstract 2

Supplemental Digital Content is available in the text.

Atrial fibrillation (AF) remains a leading contributor to cardiovascular morbidity and mortality worldwide.^1,2^ Observational studies have demonstrated an association between modifiable risk factors—specifically hypertension, obesity, alcohol consumption, and obstructive sleep apnea—and arrhythmic burden of patients with known AF.^3^ Although linked observationally, it is unclear whether modification of these risk factors may prevent new-onset AF.

Due to its high prevalence, hypertension is thought to be the single greatest contributor to the burden of AF. In population studies such as the Framingham Heart Study and Atherosclerosis Risk in Communities Study, up to 20% of AF cases are attributed to preexisting hypertension.^4,5^ Furthermore, 60% to 80% of patients with known AF have comorbid hypertension.^6^ Despite these observations, initiation of blood pressure–lowering therapy was not associated with a clear reduction in AF burden in the Framingham cohort.^7^ Similarly, a randomized comparison of the angiotensin-converting enzyme inhibitor, ramipril, versus placebo failed to demonstrate a relationship between ramipril therapy and incident AF.^8^ Secondary analyses in other studies comparing hypertensive agents (angiotensin-converting enzyme inhibitors, β-blockers [BBs], calcium channel blockers [CCBs], and diuretics) have not demonstrated a consistent benefit of one antihypertensive regimen over another for reducing AF.^9–11^ Comparisons of intensive blood pressure lowering with standard blood pressure lowering have suggested a benefit for patients with hypertension and elevated risk of cardiovascular events but not hypertension and diabetes.^12,13^ The inconsistent findings of antihypertensive therapy studies and observational studies have led some to question the strength of the direct relationship between blood pressure and AF or argue that it is driven by isolated subpopulations.^14–17^

Preventative studies on a population scale are difficult to accomplish in a randomized and adequately powered fashion with sufficient duration. To overcome this limitation, this study used a population genetics–based approach within a Mendelian randomization (MR) framework to better understand the causal role of blood pressure on the risk of AF. This technique takes advantage of the random allocation of blood pressure–associated genetic variants that occurs at conception. This random assortment minimizes the chance of environmental confounding, enabling investigation into the causal relationship between blood pressure and AF. We subsequently evaluated genetic proxies for the pathways targeted by antihypertensive medications to better understand potential class effects of antihypertensive medications on AF.

## Methods

The authors declare that all supporting data are available within the article and its Data Supplement.

### Study Populations

For the primary analysis, summary-level data for genome-wide association studies (GWAS) of hypertension and AF were used.^18,19^ Blood pressure data were obtained from the 2018 Evangelou et al International Consortium for Blood Pressure+UK Biobank GWAS meta-analysis, which included systolic blood pressure (SBP), diastolic blood pressure (DBP), and pulse pressure (PP) measurements in up to 757 601 individuals. Summary statistics for blood pressure are publicly available and were downloaded from the National Heart, Lung, and Blood Institute Genome-Wide Repository of Associations Between SNPs and Phenotypes catalog (https://grasp.nhlbi.nih.gov/FullResults.aspx). AF data (atrial flutter, paroxysmal AF, and persistent AF grouped together) were obtained from the 2018 Roselli et al AF GWAS meta-analysis from the AFGen (Atrial Fibrillation Genetics) consortium study, including 65 446 AF cases and 522 744 controls. Summary statistics for AF were contributed by the AFGen consortium (http://afgen.org), are publicly available, and may be downloaded from the Variant to Function Knowledge Portal (http://www.kp4cd.org/datasets/v2f). Because both the blood pressure exposure and AF outcome studies included participants from the UK Biobank (458 577 for BP and 351 017 for AF), bias due to sample overlap was estimated using a previously described tool, available at https://sb452.shinyapps.io/overlap.^20^ Across all ranges of sample overlap (0%–100%), there was no substantial inflation in type I error rate or bias (eg, for an observational odds ratio [OR] of 1.6 per 10-mm Hg increase in SBP with 100% exposure-outcome sample overlap, type I error remained 0.05, with bias of 0.0007).

### Study Exposures

The 2018 Evangelou et al International Consortium for Blood Pressure+UK Biobank discovery meta-analysis GWAS included up to 757 601 participants. This analysis included up to 299 024 European participants from 77 independent studies genotyped with various arrays and imputed to either the 1000 Genomes Reference Panel or the HRC platforms and 458 577 participants from the UK Biobank. Blood pressure ascertainment varied among cohorts, and study-specific details are presented in the Data Supplement.^18^ For each BP trait, genetic variants associated with SBP, DBP, and PP at genome-wide significance (*P*<5×10^−8^) were identified and linkage disequilibrium pruned using the default settings of the clump_data function of the TwoSampleMR package (distance threshold, 10 000 kb; r^2^<0.001) using the 1000 Genomes European ancestry reference panel to identify independent variants. Because the Evangelou et al^21^ study adjusted effect estimates for body mass index potentially leading to introduction of collider bias as body mass index is causal for both elevated blood pressure and AF, a sensitivity analysis was performed using systolic (n=436 419) and diastolic (n=436 424) blood pressure GWAS summary statistics from European UK Biobank participants adjusted for genotyping array, age, sex, and population structure.^21^

### Primary Outcome

The AFGen consortium identified participants from >50 studies (84.2% European, 12.5% Japanese, 2% African American, and 1.3% Brazilian and Hispanic), including participants from the UK Biobank, Biobank Japan, other international biobanks, and international cardiovascular cohort studies (adjusted for age, sex, and study-specific covariates). AF ascertainment was study specific, including diagnostic codes, electronic health record information, and self-report.

### Study Design

The primary analysis estimated the effect of blood pressure on the risk of AF using 2-sample MR with an inverse-variance weighted model with random effects. The MR-Egger bias intercept test was used to identify the presence of bias from directional pleiotropy. Sensitivity analysis was performed using weighted median MR and Egger intercept tests, which are more robust to the presence of invalid genetic instruments.^22^

Recent work has demonstrated that genetic proxies can be used to estimate the effect of individual antihypertensive drug classes on clinical outcomes using an MR framework.^23,24^ We used 2 approaches to estimate the effect of blood pressure–lowering medication on risk of AF:

1. Genes encoding the targets of antihypertensive medications (angiotensin-converting enzyme inhibitors, angiotensin receptor blockers, BBs, CCBs, and thiazide diuretic agents) were identified using DrugBank and the GeneHancer database in the GeneCards platform (v4.7).^23^ Single-nucleotide polymorphisms (SNPs) were identified within corresponding genes, promoter regions, or enhancers that were associated with SBP in the 2018 UK Biobank and International Consortium of Blood Pressure GWAS meta-analysis at genome-wide significance (*P*<5×10^−8^) and clumped to a linkage disequilibrium threshold of r^2^<0.1 using the 1000 Genomes European reference panel. These genetic variants were used as instruments to model the effect of lower SBP mediated by individual antihypertensive drug classes. The SNPs were then utilized to estimate the effect of the individual antihypertensive drug classes on the risk of AF using 2-sample inverse-variance weighted and median weighted MR as above.
2. Expression quantitative trait loci for protein targets of antihypertensive medications were used as a proxy for the action of a drug on its target (eg, variants associated with angiotensin-converting enzyme gene expression as a proxy for the angiotensin-converting enzyme inhibitor drug class).^24^ Twelve antihypertensive drug classes were considered: adrenergic neuron blocking drugs; α-adrenoceptor blockers, angiotensin-converting enzyme inhibitors, angiotensin-II receptor blockers, β-adrenoceptor blockers, CCBs, centrally acting antihypertensive drugs, loop diuretics; potassium-sparing diuretics and aldosterone antagonists, renin inhibitors, thiazides and related diuretics, and vasodilator antihypertensives. SNPs were identified for the protein targets of each drug class using the Genotype-Tissue Expression project data (release V7; dbGaP accession phs000424.v7.p2), which contain expression quantitative trait loci analyses of 48 tissues in 620 donors.^25^ SNPs defined by Genotype-Tissue Expression as the variant with the smallest nominal *P* for a variant-gene pair were selected for analysis and validated as instruments by estimating their effect on SBP using 2-sample MR. Expression quantitative trait loci with evidence of a significant effect on SBP by gene expression MR (*P*<0.05) were used for the analysis.

### Statistical Analysis

Two-sample MR was performed using the TwoSampleMR package in R (https://github.com/MRCIEU/TwoSampleMR).^26^ Variants associated with each blood pressure exposure at genome-wide significance (*P*<5×10^−8^) were harmonized with the variants from the AF GWAS^19^ and linkage disequilibrium clumped (distance threshold, 10 000 kb; r^2^=0.001) using the 1000 Genomes European ancestry reference panel, identifying a final set of independent SNPs to use as a genetic instrument for blood pressure. The exposure-outcome association was calculated for each variant independently; inverse-variance weighted 2-sample MR with random effects was used as the primary analysis with a weighted median analysis performed as a sensitivity analysis.^27^ For each variant included in the genetic instruments, the proportion of variance (R^2^) in the phenotype explained was calculated using the formula 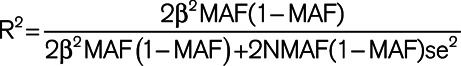 (where MAF represents the effect allele frequency, β represents the effect estimate of the genetic variant in the exposure GWAS, *se* represents the SE of effect size for the genetic variant, and N represents the sample size).^28^ F statistics were then calculated for each variant using the formula 
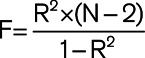
 to assess the strength of the selected instruments.^29^ Changes in blood pressure were expressed in terms of a 10-mm Hg increment as it was viewed as a clinically relatable target for blood pressure modification. All statistical analyses were performed using R, version 3.6.2.31.

## Results

### Association of Blood Pressure With AF

We identified a set of independent variants to serve as instruments for SBP (n=399) and DBP (n=398) and PP (n=347), which accounted for 4.0%, 4.2%, and 3.6% of the measured variability in these exposures, respectively (Tables S1 through S3 in the Data Supplement). For the SBP instrument, the mean F statistic was 75 (range, 30.4–645.7). For the DBP instrument, the mean F statistic was 79.9 (range, 30–846.6). For the PP instrument, the mean F statistic was 76.4 (range, 30.4–627.9). Bias due to sample overlap from the UK Biobank participants included in both the blood pressure exposure GWAS and AF outcome GWAS was estimated to be negligible across a range of observational effect sizes: for example, at 100% sample overlap, bias was estimated to be 0.0003 for an observational OR of 1.3 and 0.00069 for an observational OR of 1.6 (Table S4).

**Figure 1.**
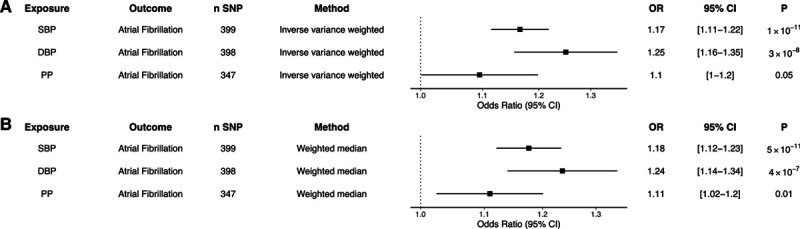
**Genetic proxies of blood pressure and risk of atrial fibrillation.** **A**, Two-sample Mendelian randomization using an inverse-variance weighted model was created using a genetic instrument associated with a 10-mm Hg increase in systolic blood pressure (SBP), diastolic blood pressure (DBP), and pulse pressure (PP) and risk of atrial fibrillation. **B**, A median weighted model was created as a sensitivity analysis. Figures are expressed as odds ratios (ORs), 95% CIs, and *P* for Mendelian randomization estimates. SNP indicates single-nucleotide polymorphism.

Two-sample MR using the above genetic instruments and inverse-variance weighted modeling demonstrated that each 10-mm Hg genetically predicted increase in SBP, DBP, and PP increased the risk of AF (SBP: OR, 1.17 [95% CI, 1.11–1.22]; *P*=1×10^−11^; DBP: OR, 1.25 [95% CI, 1.16–1.35]; *P*=3×10^−8^; PP: OR, 1.1 [95% CI, 1.0–1.2]; *P*=0.05; Figure 1). Results were similar in a sensitivity analysis using the weighted median method, with increased SBP, DBP, and PP increasing the risk of AF (SBP: OR, 1.18 [95% CI, 1.12–1.23]; *P*=5×10^−^^11^; DBP: OR, 1.24 [95% CI, 1.14–1.34]; *P*=4×10^−7^; PP: OR, 1.11 [95% CI, 1.02–1.2]; *P*=0.01; Figure 1). The effects of SBP and DBP on the risk of AF were also similar using alternative genetic instruments derived from the UK Biobank, which were not corrected for body mass index (Figure S1).

### Genetically Proxied Blood Pressure Reduction Through Antihypertensive Drug Targets and AF

**Figure 2.**
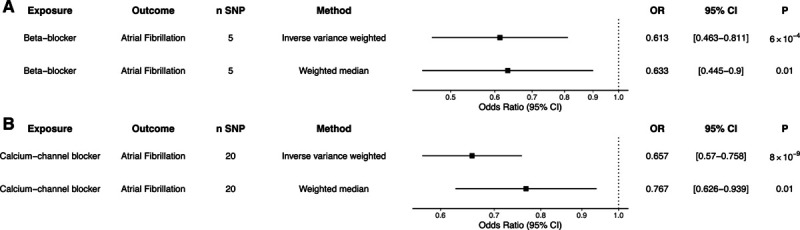
**Genetic proxies of antihypertensive medications and atrial fibrillation risk.** Two-sample Mendelian randomization was performed using genetic proxies for 10-mm Hg systolic blood pressure lowering by individual antihypertensive medication classes: (**A**) β-blockers and (**B**) calcium channel blockers. Inverse-variance weighted and median weighted models are presented. Figures are expressed as odds ratios (ORs), 95% CIs, and *P* for Mendelian randomization estimates. SNP indicates single-nucleotide polymorphism.

To estimate the effect of blood pressure reduction by different classes of antihypertensive medications, we identified common genetic variants located within genes of protein targets of CCBs and BBs, as described previously.^23^ Twenty independent variants within protein targets of CCBs and 5 independent variants within protein targets of BBs were associated with SBP at genome-wide significance (Table S5). When using these genetic proxies to estimate the effect of each 10-mm Hg decrease in SBP by each antihypertensive drug class, genetically predicted protein targets of CCBs and BBs were associated with lower risk of AF (CCB: OR, 0.66 [95% CI, 0.57–0.76]; *P*=8×10^−9^; BB: OR, 0.61 [95% CI, 0.46–0.81]; *P*=6×10^−4^; Figure 2). In a complimentary analysis, we used expression quantitative trait loci for the protein targets of antihypertensive medications given genetic variants may exert their action via distant interactions (rather than via a true *cis*-acting association; Table S6).^24^ Using this technique, antihypertensive medication proxies reduced the risk of AF (CCB: SNP, n=23; OR, 0.66 [95% CI, 0.57–0.76]; *P*=8×10^−9^; BB: SNP, n=10; OR, 0.61 [95% CI, 0.46–0.81]; *P*=6×10^−4^). There was no strong evidence of effect of other antihypertensive medication classes on AF risk (Table S7).

## Discussion

This study utilized MR to leverage population-level genetic information to explore the causal relationship between blood pressure and AF. The genetic determinants of elevated SBP and DBP were found to strongly associate with the risk of AF—an association that persisted in statistical sensitivity analyses more robust to the inclusion of pleiotropic variants. Previously validated genetic proxies for the therapeutic effects of antihypertensive drug classes were used to estimate the impact of individual antihypertensive drug classes on incident AF, suggesting a potential role for antihypertensive medications in prevention.

A relationship between hypertension and AF has previously been established in observational analyses.^4,5^ These findings, however, were limited in demonstrating a causal role for hypertension in the development of AF due to the potential of residual confounding and reverse causation.^14^ This study sought to mitigate this risk of confounding by using genetic instruments randomly assorted in the population to proxy the effect of increased blood pressure traits on the risk of AF. In doing so, we found that genetically proxied increases in SBP and DBP were associated with increased risk of AF. These elevated blood pressure effects are directionally similar to those identified in observational studies.^30^ In this analysis, PP was not as strongly associated with the risk of AF as SBP and DBP changes. As PP is dependent upon systolic and diastolic pressure, our data would suggest that the relative magnitude of each independent blood pressure parameter is more predictive than the relationship between the two. There are 2 prior studies looking at blood pressure genetics and AF; both of which are complimentary to the current study. The first was an MR analysis using an SBP polygenic risk score derived from an older blood pressure GWAS.^31^ The second used a less exhaustive set of blood pressure–related SNPS to demonstrate that SBP and DBP mediate ischemic stroke risk, in part, through AF.^32^

A variety of mechanisms have been proposed to explain how hypertension contributes to the risk of AF. Animal models of hypertension have demonstrated the presence of left atrial scaring and inflammation.^33–35^ This scaring and fibrosis is thought to create altered patterns of conduction and functional slowing, allowing for the development and perpetuation of AF triggers.^34,36^ Concordantly, hypertensive animals have greater heterogeneity of atrial activation with increased susceptibility to AF induction.^34^ Other manifestations of left atrial remodeling such as increased left atrial size have been associated with hypertension and elevated SBP in particular.^37^ It should be noted, however, that the impact of hypertension on AF risk persists after adjustment for left atrial size and mass.^30^

Beyond mechanism, this study explores the question of whether pharmacological intervention may meaningfully impact a patient’s risk of AF. While it would be ethically difficult to fully withhold antihypertensive therapy in a randomized trial, we leveraged genetic proxies to explore the impact of individual antihypertensive drug classes. Our study suggests that both CCBs and BBs can significantly mitigate a patient’s risk of developing AF. While it is tempting to compare the results of our genetic study with human pharmacological studies, there are inherent differences in these two approaches. Our analysis used genetic proxies to model antihypertensive class effects and reflects a lifetime of genetic exposure. By contrast, antihypertensive drug trials estimate the effect of only a limited duration of antihypertensive therapy initiated later in a patient’s life. It should be noted that while other drug classes like angiotensin receptor blockers did not demonstrate a significant AF-reducing effect in our study, we may have been underpowered to detect a relationship between the two due to a lack of robust genetic instruments. While it is possible that certain antihypertensive drug classes have more of an AF-reducing effect than others, our analysis is not able to fully tease out this question. It should be further noted that clinical trials and case-control analyses have not found BBs and CCBs to be consistently superior to other drug classes including angiotensin receptor blockers and angiotensin-converting enzyme inhibitors when examining impact on AF burden.^10,11,38^

A final point should be made that while the effect size of a blood pressure increase on AF was smaller in magnitude than the effect size of CCB and BB drug therapy on AF, this discrepancy may be partially explained by a nonlinear or heterogenous relationship between blood pressure reductions and blood pressure increases. The difference in effect size may also be due to the pleiotropic effects of these particular antihypertensive medications as β-receptor signaling and calcium handling have both been implicated in AF initiation and arrhythmogenesis independent of blood pressure.

### Limitations

First, while the GWAS of AF was multiethnic, the study was enriched with individuals of European ancestry as was the GWAS of blood pressure traits. This may have skewed the risk estimates in our findings, and as such, the analysis should be repeated in other populations before being generalized across ethnic groups. Second, this analysis estimates the lifelong effects of genetically predicted blood pressure reduction on AF risk and does not directly investigate the effects of shorter term alterations in blood pressure such as through pharmacological treatment in adulthood. Third, it should be noted that the risk reduction of CCBs and BBs was quantified in terms of 10-mm Hg blood pressure increments, which assumes a linear relationship. However, this study cannot answer the question of what level of blood pressure reduction maximizes AF risk reduction.

### Conclusions

Blood pressure–increasing genetic variants were associated with an increased risk of AF, consistent with a causal relationship between blood pressure and AF. These data support the concept that blood pressure reduction through pharmacological intervention and specifically calcium channel blockade or β-blockade could reduce the risk of AF.

### Perspectives

From a public health perspective, early interventions to limit lifetime exposure to elevations in SBP and DBP could have tremendous impact on the prevalence of AF. Furthermore, initiatives targeting known AF risk factors like hypertension and obesity may be key to reducing AF at a population level.

## Sources of Funding

M.C. Hyman and F.E. Marchlinski are supported by the Winkelman Family Fund in Cardiovascular Innovation. D. Gill is supported by the Wellcome Trust 4i Programme (203928/Z/16/Z) and British Heart Foundation Centre of Research Excellence (RE/18/4/34215) at Imperial College London. V.M. Walker is supported by the Medical Research Council Integrative Epidemiology Unit. The unit is supported by the UK Medical Research Council and University of Bristol (MC_UU_00011/4 and MC_UU_00011/1). N.M.D was supported by the Cohorts for Heart and Aging Research in Genomic Epidemiology (CHARGE) National Heart, Lung, and Blood Institute (NHLBI) grant R01HL105756-09 S.M. Damrauer is supported by the Department of Veterans Affairs (IK2-CX001780). This publication does not represent the views of the Department of Veterans Affairs or the US Government.

## Disclosures

All authors have completed and submitted the International Committee of Medical Journal Editors Form for disclosure of potential conflicts of interest. D. Gill is employed part-time by Novo Nordisk outside of the submitted work. S.M. Damrauer receives research support to his institution from RenalytixAI and personal consulting fees from Calico Labs, both outside the current work. The other authors report no conflicts.

## Supplementary Material



## Novelty and Significance

### What Is New?

- In this Mendelian randomization study involving summary data from >990 000 individuals, genetically predicted 10-mm Hg increases in blood pressure were associated with increased risk of atrial fibrillation (17% for systolic blood pressure and 25% for diastolic blood pressure).

### What Is Relevant?

- Analyses using genetic proxies for antihypertensive medications suggest that calcium channel blocker and β-blocker therapy may be effective in preventing atrial fibrillation.

### Summary

On a population level, blood pressure lowering may reduce the burden of atrial fibrillation.
