# Supplementary figures and images for: Genetically Predicted Blood Pressure and Risk of Atrial Fibrillation
Source: Hypertension. 2021 Jan 4;77(2):376–82. doi: 10.1161/HYPERTENSIONAHA.120.16191 (PMC7803440; doi:10.1161/HYPERTENSIONAHA.120.16191)

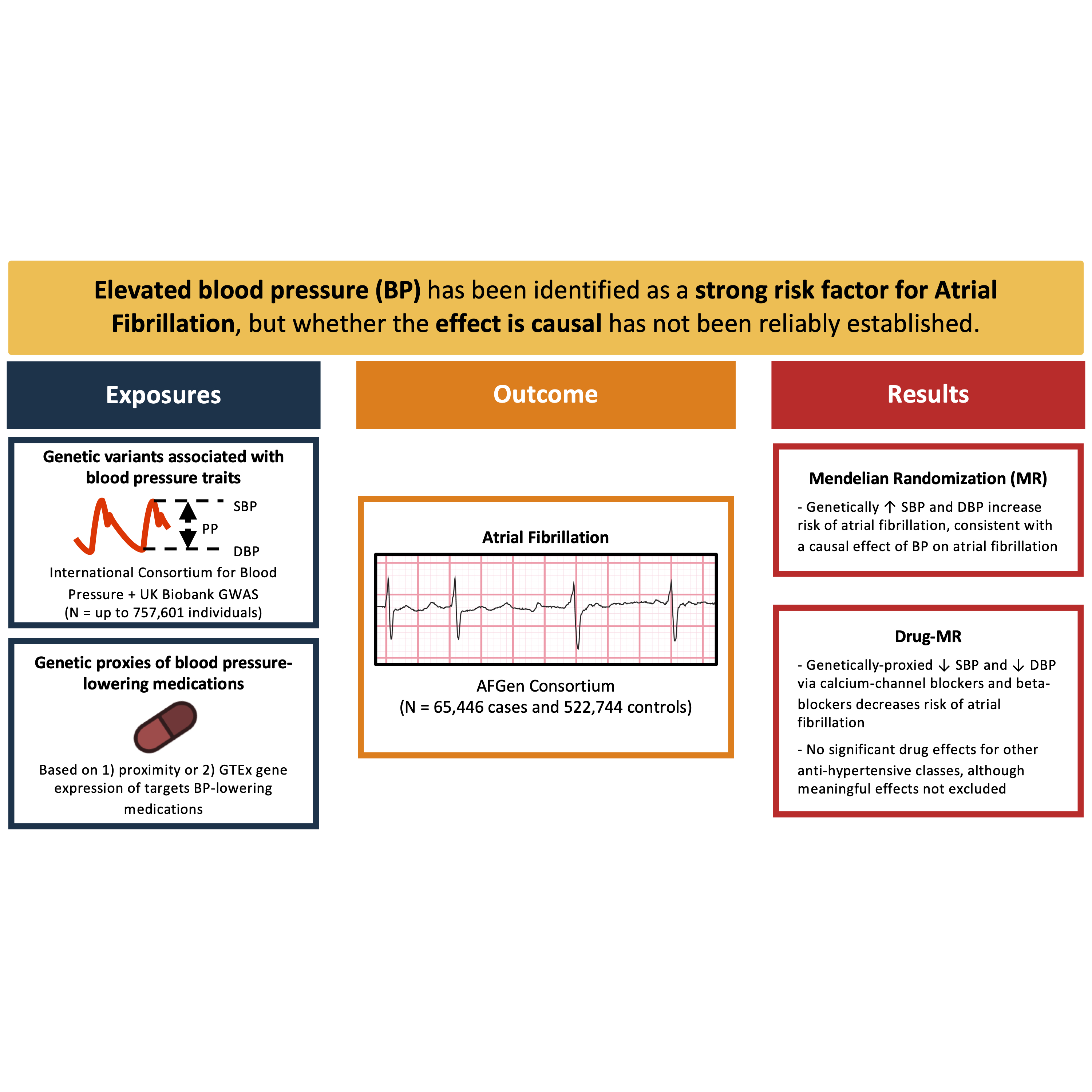

Supplement: Supplementary file 1 [file hyp-77-376-s001.jpg]
