# Supplementary material for: Genetically Predicted Blood Pressure and Risk of Atrial Fibrillation
Source: Hypertension. 2021 Jan 4;77(2):376–82. doi: 10.1161/HYPERTENSIONAHA.120.16191 (PMC7803440; doi:10.1161/HYPERTENSIONAHA.120.16191)
Supplement: Supplementary file 2 [file hyp-77-376-s002.docx]

DATA SUPPLEMENT

**Genetically Predicted Blood Pressure and Risk of Atrial Fibrillation**

Matthew C. Hyman, MD, PhD^1^*; Michael G. Levin, MD^1,2^*; Dipender Gill, BMBCh, PhD^3-7^; Venexia M. Walker, PhD^8,9,11^; Marios K. Georgakis, MD, PhD^10^; Neil Davies, PhD^8^; Francis E. Marchlinski, MD^1^ and Scott M. Damrauer, MD^2,11^

^1^Division of Cardiovascular Medicine, University of Pennsylvania Perelman School of Medicine, Philadelphia, PA
^2^Corporal Michael J. Crescenz VA Medical Center, Philadelphia, PA
^3^Department of Epidemiology and Biostatistics, School of Public Health, Imperial College London, United Kingdom

^4^Centre for Pharmacology and Therapeutics, Department of Medicine, Hammersmith Campus, Imperial College London, London, UK.

^5^Department of Genetics, Novo Nordisk Research Centre Oxford, Old Road Campus, Oxford, UK

^6^Clinical Pharmacology and Therapeutics Section, Institute of Medical and Biomedical Education and Institute for Infection and Immunity, St George’s, University of London, London, UK

^7^Clinical Pharmacology Group, Pharmacy and Medicines Directorate, St George’s University Hospitals NHS Foundation Trust, London, UK
^8^Medical Research Council Integrative Epidemiology Unit, University of Bristol, Bristol, United Kingdom

**Figure S1: Effect of Systolic and Diastolic Blood Pressure on AF Risk using UK Biobank Blood Pressure Genetic Instruments without Adjustment for Body mass Index**

**
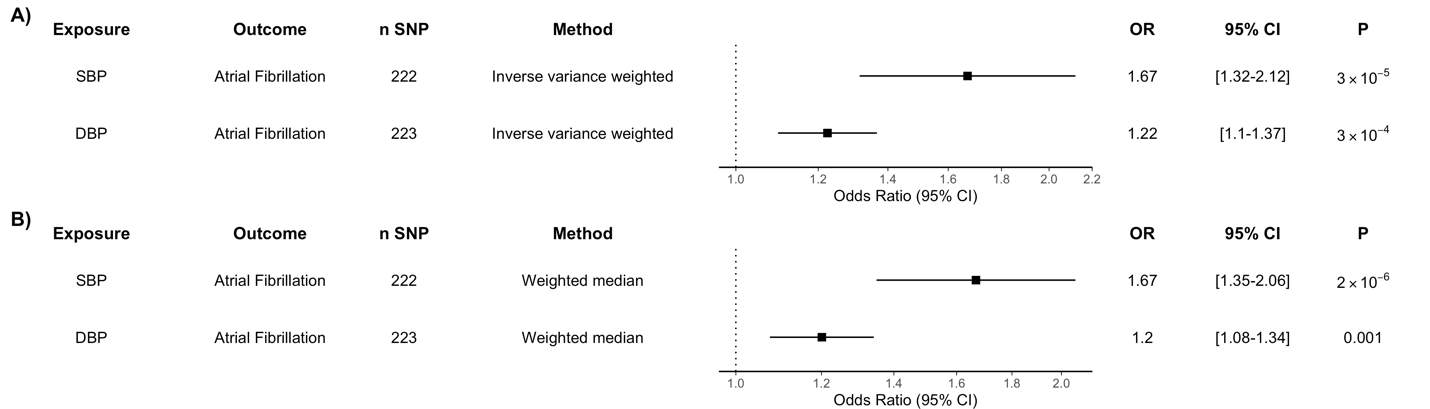
**

A) Two-sample Mendelian randomization using an inverse variance weighted model was created using a genetic instrument associated with a 10 mmHg increase in systolic blood pressure (SBP) or diastolic blood pressure (DBP) and risk of atrial fibrillation utilizing genetic instruments from the UK Biobank that were not adjusted for body mass index. B) A median weighted model was created as a sensitivity analysis. Figures are expressed as Odds Ratios (OR), 95% Confidence Intervals (CI) and P-values for Mendelian randomization estimates.
